# Traumatic brain injury patient volume and mortality in neurosurgical intensive care units: a Finnish nationwide study

**DOI:** 10.1186/s13049-016-0320-6

**Published:** 2016-11-08

**Authors:** Rahul Raj, Stepani Bendel, Matti Reinikainen, Sanna Hoppu, Teemu Luoto, Tero Ala-Kokko, Sami Tetri, Ruut Laitio, Timo Koivisto, Jaakko Rinne, Riku Kivisaari, Jari Siironen, Markus B. Skrifvars

**Affiliations:** 1 Department of Neurosurgery, University of Helsinki and Helsinki University Hospital, Topeliuksenkatu 5, PB-266, FI-00029 HUS Helsinki, Finland; 2 Division of Intensive Care, Kuopio University Hospital, Puijonlaaksontie 2, PB-100, FI-70029 KYS Kuopio, Finland; 3 Division of Intensive Care, North Karelia Central Hospital, Tikkamäentie 16, 80210 Joensuu, Finland; 4 Department of Anaesthesiology, Intensive Care and Pain Medicine, Tampere University Hospital, Teiskontie 35, PB-2000, FI-33521 Tampere, Finland; 5 Department of Neurosurgery, University of Tampere, Medical School, and Tampere University Hospital, Teiskontie 35, PB-2000, FI-33521 Tampere, Finland; 6 Division of Intensive Care, Department of Anaesthesiology, Oulu University Hospital and Oulu University, Medical Research Center Oulu, Oulu, Finland; 7 Research Group of Surgery, Anaesthesia and Intensive Care, Medical Faculty, University of Oulu, PB-22 OUH, FI-90029 Oulu, Finland; 8 Department of Neurosurgery, Oulu University Hospital, Kajaanintie 50, 90220 Oulu, Finland; 9 Department of Perioperative Services, Intensive Care and Pain Medicine, Turku University Hospital, Turku, Finland; 10 Department of Neurosurgery, Kuopio University Hospital, Puijonlaaksontie 2, PB-100, FI-70029 KYS Kuopio, Finland; 11 Department of Neurosurgery, Turku University Hospital and University of Turku, Hämeentie 11, PB-52, FI-20251 Turku, Finland; 12 Department of Anaesthesiology, Intensive Care and Pain Medicine, University of Helsinki and Helsinki University Hospital, Haartmaninkatu 4, PB-340, FI-00029 HUS Helsinki, Finland; 13 Australian and New Zealand Intensive Care Research Centre, School of Public Health and Preventive Medicine, Monash University, Melbourne, Australia

## Abstract

**Background:**

Differences in outcomes after traumatic brain injury (TBI) between neurosurgical centers exist, although the reasons for this are not clear. Thus, our aim was to assess the association between the annual volume of TBI patients and mortality in neurosurgical intensive care units (NICUs).

**Methods:**

We collected data on all patients treated in the five Finnish university hospitals to examine all patients with TBI treated in NICUs in Finland from 2009 to 2012. We used a random effect logistic regression model to adjust for important prognostic factors to assess the independent effect of ICU volume on 6-month mortality. Subgroup analyses were performed for patients with severe TBI, moderate-to-severe TBI, and those who were undergoing mechanical ventilation or intracranial pressure monitoring.

**Results:**

Altogether 2,328 TBI patients were treated during the study period in five NICUs. The annual TBI patient volume ranged from 61 to 206 patients between the NICUs. Univariate analysis, showed no association between the NICUs’ annual TBI patient volume and 6-month mortality (*p* = 0.063). The random effect model showed no independent association between the NICUs’ annual TBI patient volume and 6-month mortality (OR = 1.000, 95% CI = 0.996–1.004, *p* = 0.876). None of the pre-defined subgroup analyses indicated any association between NICU volume and patient mortality (*p* > 0.05 for all).

**Discussion and Conclusion:**

We did not find any association between annual TBI patient volume and 6-month mortality in NICUs. These findings should be interpreted taking into account that we only included NICUs, which by international standards all treated high volumes of TBI patients, and that we were not able to study the effect of NICU volume on neurological outcome.

**Electronic supplementary material:**

The online version of this article (doi:10.1186/s13049-016-0320-6) contains supplementary material, which is available to authorized users.

## Background

It is debatable whether the outcome following severe traumatic brain injury (TBI) has improved in the last 25 years [1, 2]. There are studies suggesting that TBI treatment guidelines [3, 4], aggressive neurointensive treatment regimens [5–7] and the centralization of TBI care [8] have improved patient outcomes, although the evidence is inconclusive. The centralization of trauma and TBI care to high-volume centers is arguably one of the most important steps in improving the quality of care [8, 9]. Previous studies have shown an association between higher annual TBI volume and improved survival rates in specialized trauma centers [10, 11]. Still, major differences in outcomes also exist between specialized neurosurgical centers [12]. The reasons for these inter-center differences may include variations in the patient case-mix or the quality of delivered care, and the unit patient volume quite possibly influences the latter.

The aim of the present study was to assess the independent association of annual TBI patient volume and 6-month mortality in Finnish neurosurgical intensive care units (NICU). We hypothesized that the mortality rates would be inversely associated with the NICUs’ annual TBI patient volume, thus advocating further centralization into fewer high-volume units.

## Methods

### Setting

**Fig. 1:**
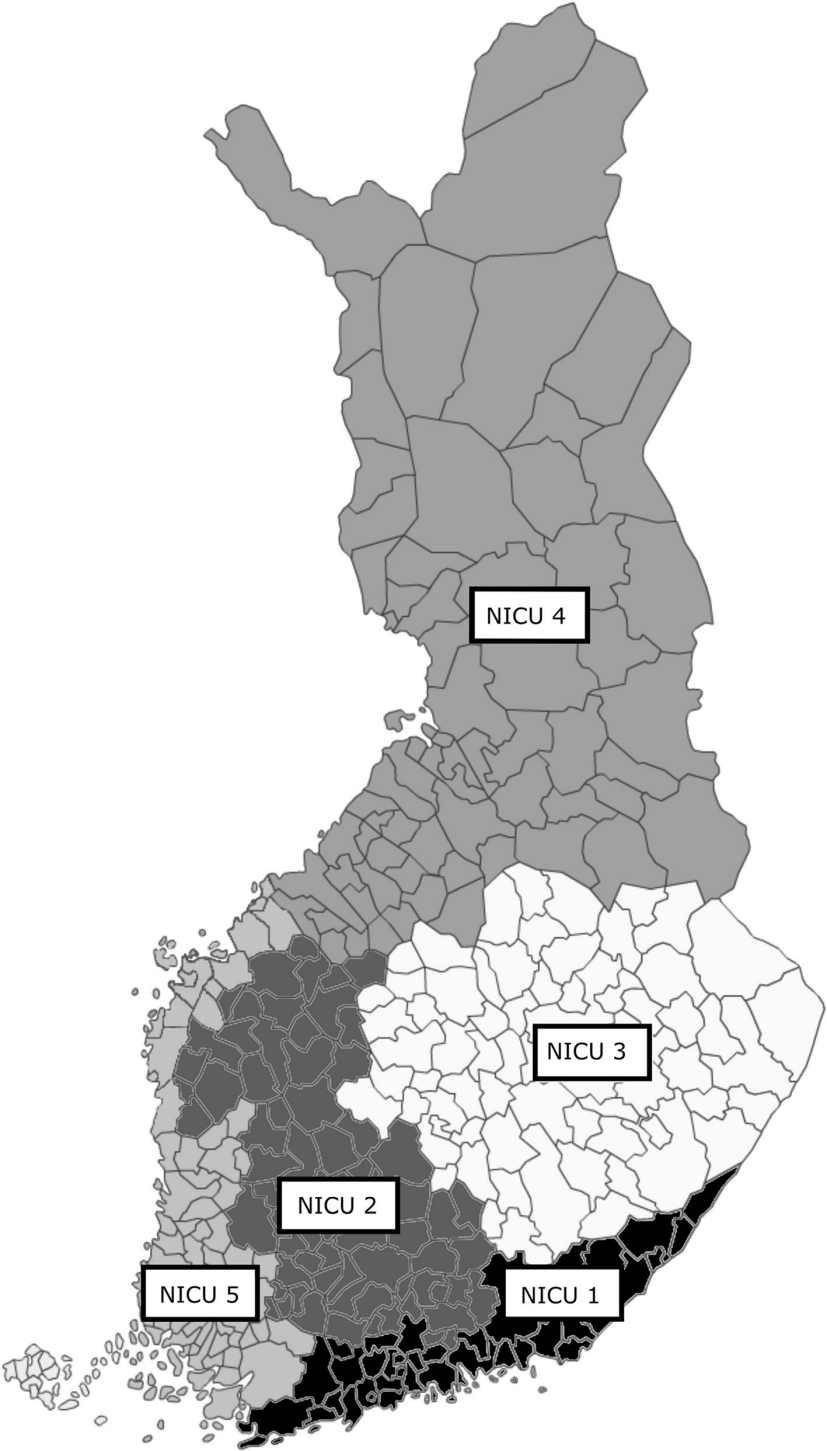
Showing the five university hospitals and their catchment area. All neurosurgical and neurointensive care is solely given in the five university hospitals’ intensive care units (NICU-1 to NICU-5 from highest to lowest annual TBI patient volume)

The Finnish healthcare system consists of a three-level system, which is publicly funded by local municipalities and the state of Finland (tax based). The healthcare system is divided into five major hospital districts (Fig. 1) that are covered at a primary level of care by local hospitals and health centers, at a secondary level of care by central hospitals and at a tertiary level of care by university hospitals. The local and central hospitals refer the patients to their own referral university hospital. There are five university hospitals in Finland (in Helsinki, Turku, Tampere, Oulu and Kuopio) covering the whole population (5,427,383 at the end of 2012, of which 4,325,139 (80%) were 18 years or older). All university hospitals are academic non-profit and publicly funded. Acute neurosurgical and neurointensive care is only provided in the five university hospitals’ ICUs (referred to as NICUs in the text). All NICUs’ treatment guidelines are based upon the Brain Trauma Foundation (BTF) guidelines of the treatment of patients with severe TBI [13].

### Study design

We conducted a nationwide open-cohort observational retrospective multi-center study using two ICU databases (Finnish Intensive Care Consortium [FICC] and a local ICU database) to cover all patients with TBI treated in NICUs in Finland. The FICC database is a nationwide prospectively data collecting ICU database in Finland, consisting of ICUs from hospitals covering the whole mainland of Finland. The FICC was founded in 1994 as a co-operative benchmarking project to improve intensive care in Finland [14]. Most of the data in the FICC are automatically collected from patient monitors and laboratory systems. The data not automatically collected (e.g., patient co-morbidity, diagnoses, vital status at hospital discharge) are manually entered by specially trained ICU personnel. All data are transferred to a central database maintained by Tieto Healthcare & Welfare Ltd (Helsinki, Finland). Before incorporation into the central database, automatic filters and specially trained personnel validate all data. The local NICU database has been previously described [15].

### Study population and data collection

We included all adult patients (age ≥18) admitted to a NICU due to TBI irrespective of their admission Glasgow Coma Scale (GCS) scores from January 1, 2009 to December 31, 2012. All included patients’ admission head computerized tomography (CT) scans were reviewed to categorize injury severity. We excluded patients with penetrating TBI (*N* = 78), re-admissions and patients with missing baseline data. If a patient was transferred between participating NICUs, only the first treatment period was included.

We combined the two databases into a joint database for the analyses. We named the NICUs from NICU-1 to NICU-5 by annual TBI volume (NICU-1 highest volume, NICU-5 lowest volume). Both databases provided data according to the Acute Physiology and Chronic Health Evaluation II (APACHE II) and Simplified Acute Physiology Score II (SAPS II) scoring systems and some baseline characteristics [16]. The Therapeutic Intervention Scoring System 76 (TISS-76) was used as a marker of treatment intensity level [17].

We extracted the patients’ admission head CT scans from the Picture Archiving and Communicating System (PACS) register of each university hospital. All CT scans were analyzed according to the Marshall CT classification jointly by the two authors (R.R., R.K.) [18]. The Marshall CT classification comprises six different classes: diffuse injury I, diffuse injury II, diffuse injury III, diffuse injury IV, non-evacuated mass lesion (defined as >25 cm^3^) and any evacuated mass lesion. Since the distinction between evacuated and non-evacuated mass lesions is artificial, we combined them into one category. We defined patient comorbidity as severe organ insufficiency (cardiovascular, liver, renal, respiratory) or significant immunocompromission according to the APACHE II methodology [16]. The GCS scores in the two databases are defined according to the SAPS II system as the worst GCS during the first 24 h in the ICU. The last reliable GCS was used for intubated and/or sedated patients.

We used 6-month all-cause mortality as the primary outcome. As a secondary outcome, we used 30-day mortality. Mortality data were extracted from the Finnish population register (available for all patients) and the archive of death certificates.

### Statistical methods

SPSS statistics for Mac, version 22.0, released 2013 (IBM Corp, Armonk, NY, USA) and R: A Language and Environment for Statistical Computing (R- Foundation for Statistical Computing, Vienna, Austria, 2013) were used for the statistical analyses.

Categorical data are presented as numbers with percentages. The *χ* ^2^ test (two-tailed) was used for categorical univariate analyses. Continuous variables were tested for skewness using the Kolmogorov–Smirnov test. Skewed data are presented as median (IQR) and were analyzed using the Mann–Whitney *U* test. Means are compared using an independent *t*-test (two groups) or a one-way analysis of variance test (more than two groups).

To assess the independent effect of the NICUs’ annual TBI patient volume on mortality, we used a random effect logistic regression model using the annual TBI volume as a continuous variable. The NICU of admission was modeled as a random effect and considered as the random part of the intercept. This structure is preferred to the traditional logistic regression analysis in order to adjust for differences in case-mix between the five NICUs, and to properly assess the independent weight of one NICU-level variable [19]. The patient-level (first level) variables were: age, GCS (defined as the worst score during the first NICU day [20]), Marshall CT classification, operative admission, severe comorbidities and annual TBI patient volume. The NICU-level (second level) variable was the NICU of admission. The area under the receiver operator curve of the multivariate analysis for predicting 6-month mortality and 30-day mortality was 0.83 (95% CI = 0.81-0.85) and 0.84 (95% CI = 0.82-0.86), respectively, indicating good discrimination and, thus, case-mix adjustment.

Subgroup analyses were performed on patients with the most severe TBI, defined as the worst 24-h GCS of 3 to 8; patients with moderate-to-severe TBI (GCS 3 to 12); patients requiring mechanical ventilation; and patients undergoing ICP-monitoring. The results are presented as odds ratios (OR) with 95% confidence intervals (CI). Due to the large patient number, we defined *p*-values <0.01 as statistically significant.

The study was performed according to the STROBE recommendations (Additional file 1) [21].

## Results

Altogether 2,328 TBI patients were treated during the study period in the five NICUs. NICU-1 treated on average 206 patients annually, NICU-2 treated 122 patients, NICU-3 treated 111 patients, NICU-4 treated 82 patients and NICU-5 treated 61 patients. Regarding moderate-to-severe TBIs, NICU-1 treated on average 137 patients annually (67% of all), NICU-2 69 patients (57% of all), NICU-3 65 patients (59% of all), NICU-4 55 patients (67% of all) and NICU-5 44 patients (72% of all).

Differences in baseline patient characteristics between the NICUs are shown in Table 1. The median patient age was 58 years (IQR = 45-68). Patients in NICU-4 had a somewhat lower median age than in the other ICUs, and a larger proportion of the patients in NICU-4 had a GCS of 8 or lower. The presence of a large mass lesion (>25 cm^3^) was more frequently observed in NICU-1 than in the other NICUs (64% vs. 42–49%). Operative admission ranged from 29% to 42% between the NICUs.

**Table 1:** Study population baseline characteristics

	NICU-1 (*N* = 206/year)	NICU-2 (*N* = 122/year)	NICU-3 (*N* = 111/year)	NICU--4 (*N* = 82/year)	NICU-5 (*N* = 61/year)	*p*-Value
Baseline characteristics						
Age, median (IQR)	58 (46-68)	58 (45-70)	59 (49-69)	53 (35-65)	59 (44-67)	<0.001
< 45	191 (23)	122 (25)	82 (18)	108 (33)	63 (26)	0.001
45-75	517 (63)	285 (58)	293 (66)	183 (56)	148 (61)	
> 75	117 (14)	82 (17)	70 (16)	36 (11)	31 (13)	
Severe co-morbidities*	55 (7)	37 (8)	45 (10)	35 (11)	14 (6)	0.046
GCS, median (IQR)	10 (6-13)	11 (6-14)	11 (7-14)	8 (3-14)	9 (5-13)	<0.001
3-8	351 (43)	178 (36)	156 (35)	166 (51)	118 (49)	<0.001
9-12	198 (24)	97 (20)	104 (23)	52 (16)	56 (23)	
13-15	276 (34)	214 (44)	185 (42)	109 (33)	68 (28)	
Operative admission	308 (37)	169 (35)	127 (29)	136 (42)	91 (38)	0.002
Marshall CT						
DI I	9 (1)	48 (10)	24 (5)	40 (12)	21 (9)	<0.001
DI II	229 (27)	175 (36)	158 (36)	112 (34)	85 (35)	
DI III	32 (4)	45 (9)	42 (9)	33 (10)	17 (7)	
DI IV	30 (4)	7 (1)	3 (1)	5 (2)	1 (0)	
EML or NEML	525 (64)	214 (44)	218 (49)	137 (42)	118 (49)	<0.001
APACHE II, median	15 (11-19)	16 (10-23)	16 (10-23)	19 (12-25)	19 (12-24)	<0.001
Treatment variables						
Mechanically ventilated	668 (81)	256 (52)	231 (52)	239 (73)	207 (86)	<0.001
ICP monitored	138 (17)	74 (15)	83 (19)	117 (36)	60 (25)	<0.001
Lowest MAP, mmHg^†^	59 (50-67)	70 (64-78)	66 (59-75)	67 (60-74)	66 (60-72)	<0.001
Lowest PaO_2_, kPa^‡^	14.4 (11.4-19.5)	11.3 (9.7-13.2)	12.4 (10.2-16.2)	12.6 (10.3-15.6)	13.2 (10.7-16.7)	<0.001
Length of stay (days)						
Intensive care unit	2.3 (1.0-6.0)	1.4 (1.0-3.1)	1.2 (0.9-2.4)	2.0 (1.0-5.0)	2.9 (1.4-6.2)	<0.001
Hospital	8.0 (4.0-14.0)	5.0 (2.0-13.0)	4.0 (3.0-7.0)	7.0 (4.0-12.0)	8.0 (4.0-13.0)	<0.001
TISS-76^§^						
Mean score	32 (26-37)	25 (21-31)	22 (17-30)	28 (21-35)	31 (25-35)	<0.001
Total score	106 (50-259)	61 (40-121)	50 (31-79)	82 (41-219)	120 (57-249)	<0.001
Observed mortality						
30-day	139 (17)	84 (17)	76 (17)	49 (15)	59 (24)	0.046
6-month	195 (24)	108 (22)	92 (21)	58 (18)	66 (27)	0.063
Mean (SD) predicted risk for death^¶^						
30-day	16.8 (17.4)	17.2 (20.8)	17.1 (19.3)	15.0 (17.1)	24.4 (22.6)	<0.001
6-month	23.6 (20.9)	22.1 (23.5)	20.7 (21.4)	17.7 (19.4)	27.3 (23.7)	<0.001

Continuous data are shown as median (IQR) and categorical data are shown as absolute numbers with percentages (%)

*Abbreviations*: DI, Diffuse Injury; GCS, Glasgow Coma Scale (worst 24-h score); APACHE II, Acute Physiology and Chronic Health Evaluation II; NICU, Neurosurgical Intensive Care Unit; EML, Evacuated Mass Lesion; NEML, Non-Evacuated Mass Lesion; MAP, Mean Arterial Pressure; PaO_2_, arterial oxygen tension; ICP, Intracranial Pressure; TISS-76, Therapeutic Intervention Scoring System 76

*Defined according to the APACHE II criteria as severe organ dysfunction. †Available for 2,187 of 2,328 patients. ‡Available for 2,249 of 2,328 patients. §Available for 2,300 of 2,328 patients. ¶Using a standard logistic regression model adjusting for age, GCS, Marshall CT, severe comorbidity and treatment hospital

Marshall DI I indicates a normal admission head CT scan, Marshall DI II indicates any traumatic pathology with midline shift 0-5 mm, normal basal cisterns and no mass lesion >25 cm^3^, DI III indicates compressed or obliterated basal cisterns with midline shift 0-5 mm and no mass lesion >25 cm^3^, DI IV indicates midline shift >5 mm with no mass lesion >25 cm^3^, EML or NEML any mass lesion >25 cm^3^

A notably larger proportion of patients underwent mechanical ventilation in NICU-1 (81%), NICU-4 (73%) and NICU-5 (86%) than in NICU-2 (52%) and NICU-3 (52%). Monitoring of the ICP was the most frequent in the smaller volume NICU-4 and NICU-5 compared to in the other NICUs (36% and 25% compared to 15–19%). Of patients with a day one GCS of 8 or lower, 30% were ICP monitored (at some point during their stay) in NICU-1, 34% in NICU-2, 41% in NICU-3, 58% in NICU-4 and 32% in NICU-5 (*p* < 0.001). The NICU and hospital stays were longer in NICU-1 and NICU-5 than in the other NICUs. Correspondingly, the total treatment intensity was higher in these NICUs, while no major difference in mean treatment intensity levels were noted. There were some differences in MAP and PaO_2_ between the NICUs, but the absolute differences were small with overlapping interquartile ranges (Table 1).

### Intensive care unit volume and mortality

Overall unadjusted 6-month and 30-day mortality rates were 22% (*N* = 519) and 18% (*N* = 407), and did not significantly differ between the NICU volumes (*p* = 0.550 and *p* = 0.507, respectively). The mean predicted risks for 30-day and 6-month mortality are presented in Table 1. Regarding patients with moderate-to-severe TBI, no statistically significant differences in unadjusted 6-month mortality (range 29–32%, *p* = 0.572) or 30-day mortality (range 23–26%, *p* = 0.452) were found between the NICU volumes.

Including all patients (mild-to-severe TBI), the random effect model showed no independent association between NICU volume and 6-month mortality (OR = 1.000, 95% CI = 0.996-1.004, *p* = 0.876) or 30-day mortality (OR = 0.997, 95% CI = 0.993–1.000, *p* = 0.114).

With regards to subgroup analyses, no statistically significant associations were found between NICU volume and 6-month mortality in patients with a GCS score between 3 and 12 (OR = 1.000, 0.994–1.006, *p* = 0.967); GCS score of 8 or lower (OR = 0.999, 95% CI = 0.993-1.004, *p* = 0.663); patients mechanically ventilated (OR = 0.999, 95% CI = 0.995–1.003, *p* = 0.619); patients undergoing ICP monitoring (OR = 0.999, 95% CI = 0.994–1.003, *p* = 0.589). Similar results were found regarding 30-day mortality for all subgroups (OR = 1.00, 95% CI = 0.99-1.00, *p* = 0.429 for GCS 3-12; OR = 1.00, 95% CI = 0.99-1.00, *p* = 0.240 for GCS ≤8; OR = 1.00, 95% CI = 0.99-1.00, *p* = 0.176 for mechanically ventilated patients; OR = 1.00, 95% CI = 0.99-1.00 *p* = 0.287 for ICP-monitored patients).

## Discussion

In this nationwide register-based observational multi-center study, we investigated TBI patient volume-associated differences in the mortality of patients with TBI treated in NICUs in Finland. After adjusting for important prognostic factors and accounting for random variations, we found no independent association between TBI patient volume and mortality. Subgroup analyses in patients with the most severe TBI (GCS 3 to 8 or 3 to 12, mechanically ventilated patients, ICP-monitored patients) yielded similar results. These findings indicate that further centralization of TBI patients into high-volume units does not necessarily improve patient survival in already high-volume NICUs. It should be emphasized, that the NICU with the lowest annual TBI patient volume still treated more than 60 patients annually, which by international standards might be seen as high. Thus, one should be cautious to generalize our results to units treating fewer patients than this annually.

Higher hospital patient volume seems to be inversely associated with patient mortality after high-risk surgery [22]. This may be due to increased treatment experience and the availability of appropriate complementary medical, radiological and surgical support and services (ICP-monitoring possibilities, craniotomy expertise, etc. in the case of TBI). Additionally, in complex elective surgery, individual surgeon operation volume is an important determinant of patient outcomes, independent of hospital volume [23]. In the ICU, the ratio of the number of patients treated to ICU beds, the ratio of ICU beds to hospital beds and the nurse to patient ratio are further factors that have been previously shown to affect outcomes in mixed cohorts of ICU patients [24, 25]. In TBI patients, several studies have shown outcome benefits of treating TBI in specialized neurosurgical trauma centers [8, 26, 27]. However, the association between hospital or NICU volume and outcomes after TBI is rather scarce, although there are some studies suggesting improved outcomes in high-volume centers [10, 11, 28]. Securing appropriate TBI care for the whole population while minimizing costs and maintaining equally good patient outcomes should be a goal for countries around the world. Our results suggest that centralization into units with very high volumes does not necessarily improve survival.

The reported overall 6-month mortality for patients with moderate-to-severe TBI (GCS 12 or under) in our study was 31%, which is comparable to previous observational series (32–46% [29, 30]). No significant association between unadjusted 30-day or 6-month mortality and NICU volume were found. It is, however, inadequate to directly compare unadjusted mortality rates as such a comparison does not account for inter-center differences in the case mix. To adjust for patient case mix, we used a random effect logistic regression model, adjusting for previously known predictors of outcomes in TBI [20, 31]. The adjustment is only as good as the included variables and this methodology does not exclude, for example, population and geographical differences, something that has to be considered in a nationwide study such as this. For example, in the Eastern parts of Finland, the unemployment and alcohol consumption rates are the highest in the country, whereas the degree of education is the lowest (data from https://www.sotkanet.fi/sotkanet/en/index?, Statistical Information of Welfare and Health in Finland), all factors that might predispose to TBI and poorer outcome, possibly affecting our results [32]. Also, as shown in Fig. 1, some of the smaller units cover a relatively large proportion of the rural Finnish area. It is known that rural areas with low population density and longer transport distances have a larger proportion of pre-hospital deaths [33]. According to the statistics of the Ministry of Social Affairs and Health in Finland, sparsely populated Northern and Eastern Finland have higher trauma mortality rates compared to the densely populated Southern Finland [34]. This might contribute to a less severely injured TBI population reaching the NICUs covering these areas, influencing our results.

It is plausible that age and GCS are the strongest outcome predictors in patients with TBI [31]. We used the patients’ worst GCS measured in the first 24 h in the ICU, which might be a stronger predictor of outcome than admission GCS. TBI patients are often intubated and/or sedated during the first 24 h, and thus, using the worst 24 h GCS might cause some distortion. For these patients, however, the pre-sedation and/or pre-intubation GCS scores were used. This might have affected our results, as more patients in the lowest volume ICU had a GCS ≤8 while having lower Marshall CT classes. Thus, these patients might have been more heavily sedated, giving them a lower GCS score and masking potentially better outcomes in the higher volume NICUs.

Although we did not analyze treatment differences between the NICUs per se, some treatment features deserve mentioning. We analyzed the lowest measured mean arterial pressure (MAP) and arterial oxygen tension (PaO_2_), which may be considered as indirect surrogate markers for cerebral perfusion pressure and partial brain tissue pressure. The absolute differences in MAP and PaO_2_ were small between the NICUs with overlapping confidence intervals, suggesting no major differences in these. Monitoring of the ICP was more frequently performed in the lower volume NICUs and although all NICUs’ treatment guidelines are based upon the BTF guidelines surprisingly few patients with severe TBIs (approximately 40 %) were ICP-monitored. Monitoring of the ICP has not been shown to improve outcome, and the observed differences in ICP monitoring is probably due to inter-unit differences in the case mix of patients [35]. This is supported by the fact that the lower volume NICUs had significantly more patients with a GCS of 8 or lower, and more patients were receiving mechanical ventilation. Thus, although this might reflect treatment strategy differences between the NICUs it might also be a confounding factor something that we cannot control given the study design. It should be emphasized that the GCS was defined as the worst GCS measured in the first 24 h. Some patients with a worst GCS of 8 or lower might have had a best GCS higher than 8, thus, not warranting ICP-monitoring. There were no major differences in mean treatment intensity level between the different NICU volumes, although the total treatment intensity levels (which directly correlates with NICU length of stay) were higher in some NICUs, indicating more severely ill TBI patients in the units with longer lengths of stay, further supporting differences in the patient case mix. In some of the units, a large proportion of the mild TBI patients were mechanically ventilated. This might be because some patients were mechanically ventilated for extra-cranial causes or because patient level of consciousness at a later stage in the ICU mandated sedation and mechanical ventilation (before or after the first treatment day).

### Limitations

There are some limitations to our study that must be acknowledged. First, due to the retrospective nature of the study, we were limited to using mortality as the primary end-point and could not determine neurological outcome, which is a highly relevant outcome measure in TBI patients. Second, we were only able to account for differences in NICU parameters and were unable to adjust for differences in post-ICU discharge. It is possible that there are differences in the quality of care in the step-down rehabilitation hospitals, where the TBI patients are sent from the university hospitals, which might affect our results. Future studies should evaluate the entire treatment chain, from the pre-hospital care to the rehabilitation units, the latter often being neglected in TBI research. Third, as we only included patients admitted to the NICU, our study does not account for the most severe TBI patients dying prior to hospital admission or in the emergency departments, something that may skew our results, considering that pre-hospital transport distances vary greatly between the five hospital districts in Finland (see Fig. 1). Fourth, the FICC does not include data on pupillary reactivity, a prognostic factor that is highly relevant in TBI patients [36]. However, previous studies have shown that a prognostic model based only upon age and GCS is accurate for predicting mortality in TBI patients, even in the absence of pupillary reactivity [20, 37]. Fifth, the FICC database does not include data on overall injury severity measures, such as the injury severity score. This might contribute to the relative large proportion of mild TBIs in some of the units. However, the additional prognostic value of extra-cranial injuries in severe TBI patients is minimal and would probably not have changed our results [15].

## Conclusion

We did not find any association between annual TBI patient volume and 6-month mortality in NICUs. These findings should be interpreted taking into account that we only included NICUs, which by international standards all had high volumes of TBI patients, and that we were not able to study the effect of ICU volume on neurological outcome.

## Additional file

Additional file 1: STROBE Statement. Checklist of items that should be included in reports of observational studies. (DOC 104 kb)
